# Chitosan versus Carboxymethyl Chitosan Cryogels: Bacterial Colonization, Human Embryonic Kidney 293T Cell Culturing and Co-Culturing

**DOI:** 10.3390/ijms232012276

**Published:** 2022-10-14

**Authors:** Andrey Boroda, Yuliya Privar, Mariya Maiorova, Irina Beleneva, Marina Eliseikina, Anna Skatova, Dmitry Marinin, Svetlana Bratskaya

**Affiliations:** 1 A.V. Zhirmunsky National Scientific Center of Marine Biology, Far Eastern Branch of Russian Academy of Sciences, 17 Palchevskogo St., 690041 Vladivostok, Russia; 2 Institute of Chemistry, Far Eastern Branch of the Russian Academy of Sciences, 159 Prosp.100-Letiya Vladivostoka, 690022 Vladivostok, Russia

**Keywords:** scaffolds, cryogels, 3D culturing, multicellular spheroids, HEK-293T cell line, scanning electron microscopy, flow cytometry

## Abstract

The potential of chitosan and carboxymethyl chitosan (CMC) cryogels cross-linked with diglycidyl ether of 1,4-butandiol (BDDGE) and poly(ethylene glycol) (PEGDGE) have been compared in terms of 3D culturing HEK-293T cell line and preventing the bacterial colonization of the scaffolds. The first attempts to apply cryogels for the 3D co-culturing of bacteria and human cells have been undertaken toward the development of new models of host–pathogen interactions and bioimplant-associated infections. Using a combination of scanning electron microscopy, confocal laser scanning microscopy, and flow cytometry, we have demonstrated that CMC cryogels provided microenvironment stimulating cell–cell interactions and the growth of tightly packed multicellular spheroids, while cell–substrate interactions dominated in both chitosan cryogels, despite a significant difference in swelling capacities and Young’s modulus of BDDGE- and PEGDGE-cross-linked scaffolds. Chitosan cryogels demonstrated only mild antimicrobial properties against *Pseudomonas fluorescence*, and could not prevent the formation of *Staphylococcus aureus* biofilm in DMEM media. CMC cryogels were more efficient in preventing the adhesion and colonization of both *P. fluorescence* and *S. aureus* on the surface, demonstrating antifouling properties rather than the ability to kill bacteria. The application of CMC cryogels to 3D co-culture HEK-293T spheroids with *P. fluorescence* revealed a higher resistance of human cells to bacterial toxins than in the 2D co-culture.

## 1. Introduction

One of the key issues in the engineering of biomaterials is the need for the development of dual- or multi-functional scaffolds which, aside from supporting cell proliferation and tissue regeneration, can prevent bacterial growth [1,2,3,4] or possess hemostatic properties [5]. This target in most cases is achieved by a combination of strategies, including the careful selection of the polymer or polymer blends, the incorporation of antimicrobial proteins [6] or silver nanoparticles [3,7], and surface patterning [8]. The high interest to the application of chitosan and its derivatives in cell culturing and biomedicine is determined by their structural similarity to glycosaminoglycans and, thus, to extracellular matrix [9], as well as hemostatic [5], pro-angiogenic [10], and antimicrobial properties [11,12,13]. Despite a very broad spectrum of chitosan derivatives reported in the scientific literature, there are only few commercially available ones, which can be used to scale-up designed multifunctional biomaterials. Carboxymethyl chitosan (CMC) is the first and still the most popular type of chitosan derivative, whose synthesis has been transferred from the lab to industry and commercialized. In comparison with chitosan, CMC is soluble at neutral and alkaline media, more hydrophilic, and suitable for the fabrication of superabsorbents [14].

3D polymer scaffolds, which provide structural and mechanical support for cells and mimic cell–cell and cell–substrate interactions in vivo, are in high demand for cell therapy, tissue engineering, wound healing, in vitro drug testing, etc. Although hydrogels have been broadly and successfully used for cell encapsulation and culturing, macroporous scaffolds with pore sizes above 10 µm attract increasing attention. They provide the efficient transport of nutrients and cell metabolites, large inner space for multicellular structures growth, and the possibility to investigate fluid flow effect on cell–cell and cell–matrix interactions in various 3D models, which can replace animal tests and provide better correlation between in vitro and in vivo results than 2D cell cultures [15,16,17,18,19]. Both chitosan and CMC cryogels have been used for the fabrication of scaffolds for cell culturing, wound healing, and tissue engineering [20,21,22,23], but the direct comparison of their performance under the same conditions is not a common practice. In most studies involving several types of materials, chitosan or CMC were compared with other types of biopolymers or synthetic polymers [21,22], when the choice of chitosan or CMC was motivated mainly by biodegradability, non-toxicity, and the ability of both polymers to act as reducing agents and stabilizers for antimicrobial nanoparticles.

Recently, we have demonstrated that the micro-tumor morphology can be controlled by choosing the polymer for the scaffold fabrication: CMC-based cryogels have stimulated the formation of HCT 116 spheroids, while chitosan cryogels supported the cell–matrix interaction and guided HCT 116 to grow as epithelial-like continuous sheets [24]. Here we were aimed to extend comparative evaluation of the potential of chitosan and CMC cryogels to mimic the physiological function of the extracellular matrix, support multicellular structure growth, and prevent bacterial colonization of the surface that is crucial for the scaffold’s application in wound healing, tissue engineering, and the development of 3D models of bioimplant-associated infections.

A non-cancerous human embryonic kidney cell line (HEK-293T) was selected, since it is one of the most widely studied cellular model for different purposes, including manufacturing therapeutic proteins and viral vectors for research and pre-clinical studies [25,26,27], the evaluation of the selectivity [28] and nephrotoxicity [29] of anticancer drugs, and the efficiency of cell protective therapy [29]. The HEK-293T line was also used to study human cell–bacteria interactions [6,30] in order to examine the ability of *Staphylococcus aureus* to cross the epithelial barrier, transmigrate into deeper sections of host tissues, persist, and replicate intracellularly, causing cell death.

## 2. Results

### 2.1. Fabrication and Characterization of Cryogels

Type and degree of cross-linking are among the major factors which determine the porosity, swelling, and mechanical properties of cryogels and, thus, significantly affect their performance as scaffolds for cell culturing. Taking into account that large pores are beneficial for nutrient and metabolite transport and provide more space for the cellular spheroid’s growth, scaffold fabrication conditions were selected to obtain mechanically stable, elastic, and permeable cryogels with larger pore sizes in comparison with materials earlier reported by our group for HCT 116 cell culturing [31]. Since the lower molecular weight of the polymer, lower polymer and cross-linker concentrations, and higher freezing temperatures are known to promote formation of larger pores [19,32], we have selected low molecular weight chitosan and CMC, and set the polymer concentrations to 3% and the cryogelation temperature to −10 °C. Prior to thawing, the solutions were kept frozen longer than was required for the chemical cross-linking (7 days for CMC and 12 days for chitosan) to improve mechanical properties of cryogels, as was demonstrated for hyaluronic acid cryogels [32] and corroborated in our experiments [31].

For the comparison with cryogels cross-linked with BDDGE [31], we have selected as a cross-linker PEGDGE with Mn~500. This was expected to allow the fabrication of chitosan and CMC cryogels with a broader variation of elasticity, swelling, and pore size distribution to distinguish the effects of polymer nature and the scaffold’s physical characteristics on bacterial adhesion/colonization and 3D cell culturing.

Since the high swelling capacity of PEG blocks resulted, in our previous study, in the loss of permeability of chitosan cryogels with high cross-linking density [33], the lowest PEGDGE concentration corresponding to a PEGDGE:chitosan molar ratio of 1:20 was tested first and yielded a permeable cryogel with high swelling capacity. However, mechanically stable CMC cryogels in 3% polymer solutions could be obtained only at a higher PEGDGE concentration corresponding to the PEGDGE:CMC molar ratio of 1:8. At this PEGDGE:polymer concentration, the chitosan-based cryogel lost permeability due to the high swelling of the polymer phase and low pore volume.

The FTIR spectra of chitosan and CMC cryogels cross-linked with BDDGE and PEGDGE, in comparison with the original polymers (Figure S2, Supplementary Materials), showed an increase in the relative intensity of the peak at ~1000–1200 cm^−1^, attributable to the C−OH and C−O−C bonds [34,35], and at 3000–3600 cm^−1^ belonging to the hydroxyl groups and water bound to PEG and BD blocks. The higher relative intensity of this broad peak for the CH-PEG and CMC-PEG cryogels confirms the high efficiency of PEGDGE as a cross-linker and higher hydrophilicity of these scaffolds. It should be also mentioned that peaks at ≈911 and 839 cm^−1^ characteristic for the epoxy ring [10] were not detected.

**Figure 1:**
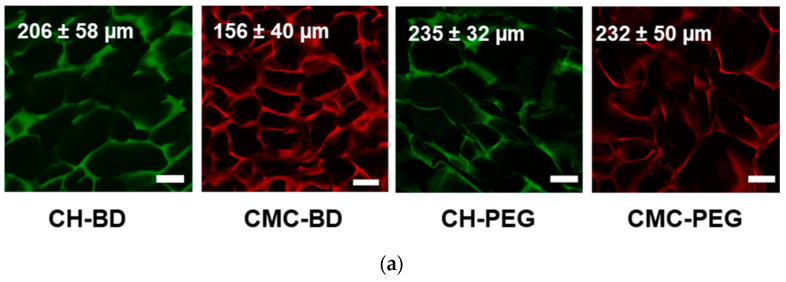

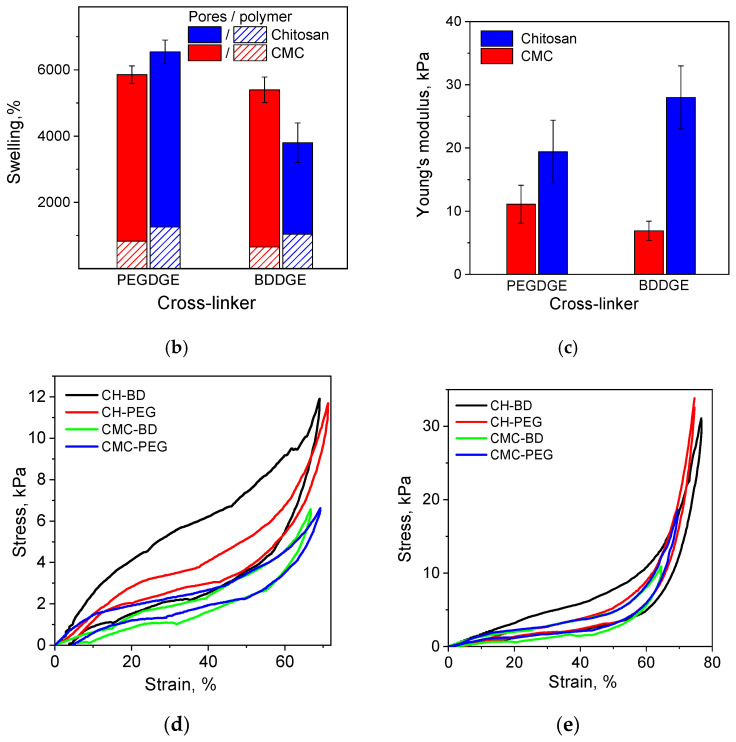
Characteristics of CMC- and chitosan (CH)-based cryogels cross-linked with 1,4-butanediol diglycidyl ether (CH-BD and CMC-BD) and poly(ethylene glycol) diglycidyl ether (CH-PEG and CMC-PEG): Confocal laser scanning microscopy (CLSM) images and average pore sizes, scale bar—100 µm (**a**); swelling (**b**), Young′s moduli (**c**) and strain–stress curves measured in the air (**d**) and in contact with water (**e**).

Figure 1 shows that, in comparison with earlier reported BDDGE-cross-linked materials [31], chitosan and CMC cryogels cross-linked with PEGDGE under selected here conditions had larger average pore size (235 ± 32 and 232 ± 50 µm, respectively) and larger swelling capacity. The interconnected porous structure of all materials allowed free water outflow under compression and reabsorption after removing the pressure. All cryogels recovered their initial size and geometry after compression/swelling cycle (Figure S3, Supplementary Materials), however, their permeability and mechanical properties significantly depended on the nature of the polymer and cross-linker. Young’s moduli (Figure 1c), calculated for the compression strain up to 14%, did not illustrate this difference in the cryogels’ behavior, so dynamic uniaxial compression–strain tests were performed on completely swollen cryogels up to 70–75% compression in two modes: samples were placed at the measuring platform of the rheometer (Figure 1d and Figure S3, Supplementary Materials) or immersed in Petri dish with a thin water layer, which was mounted at the measuring platform (Figure 1e).

Hysteresis under a cyclic load showed two distinct pathways in the loading and unloading parts of stress–strain curves for all materials in both measuring modes, and revealed significant differences in the elastic properties of the materials. First of all, chitosan-based cryogels had higher compressive strength and larger hysteresis than CMC-based cryogels, regardless of the type of cross-linker and measuring mode. Second, the largest hysteresis, i.e., the largest amount of energy lost during compression, was found for chitosan cryogel cross-linked with BDDGE, which had the highest compressive strength. The application of PEGDGE as a cross-linker at a much lower molar ratio to chitosan (1:4 for BDDGE and 1:12 for PEGDGE) yielded a cryogel with the same maximal compressive strength of 11 kPa (Figure 1d), but with more elastic behavior (lower hysteresis between loading and unloading paths). Third, measuring stress–strain curves on cryogels in contact with water showed lower hysteresis and significant increase of the compressive strength in comparison with measurements in the air. The difference was more notable for PEGDGE-cross-linked cryogels. This behavior correlates with the intrinsic properties of cryogels, whose mechanical properties in the swollen state are mainly determined by the swelling pressure and capillary forces [36].

It is worth mentioning that the selection of PEGDGE as a cross-linker enabled one to fabricate CMC cryogels with larger pore size and swelling capacity without loss in mechanical strength. This may be related to the higher cross-linking efficiency of PEGDGE in comparison with BDDGE under the selected conditions. The poor control of CMC hydrogels composition by the amount of PEGDGE, when cross-linking was performed at 60 °C [37], suggests that the undesired grafting of PEGDGE to the side chain instead of cross-links formation is a typical problem in scaffold fabrication using diglycidyl ethers.

### 2.2. Culturing HEK-293T Cells in Adhesive, Ultra-Low Attachment Conditions and 3D Scaffolds

HEK-293T cells attached to the polystyrene culturing plates within 24 h of seeding. The lag phase, when cells did not proliferate with little decrease in cell viability, took 2 days (Figure S4, Supplementary materials). The cells grew exponentially until the 7th day, covering all available surfaces and reaching the highest cell density of 300–400 × 10^3^ cells/cm^2^, however, with an abrupt decrease in cell activity from about 90% (at 4th day) to about 35% (at 7th day). Cell–substrate contacts inhibition and overgrowth resulted in the degradation of cell culture by the 14th day, with an ongoing decrease in cellular activity to 20% and density to about 180–200 × 10^3^ cells/cm^2^. These observations are considered to be normal for HEK-293T cells that demand subcultivation one to two times per week, as recommended by the manufacturer (https://www.abcam.com/human-wild-type-hek-293t-cell-line-ab255449.html, accessed on 20 July 2021). Moreover, in attempts to design an efficient nutrient medium for HEK-293 cells, the longest time before the beginning of cell culture degradation was about 192 h (8 days) [38].

Seeding HEK-293T cells into ultra-low attachment plates resulted in cell aggregation. Lag phase, similar to adhesive culturing conditions, were observed until the 3rd day. Cell activity was at a high level of about 80–95%. Then, the round-shaped spheroids of hundreds of cells in each were formed (Figure S4, Supplementary Materials). As this cell line is adherent, the lack of the cell contacts with a substrate resulted in a significant inhibition of cell proliferation. Spheroids aggregated to each other and continued to grow, forming cellular conglomerates of millions of cells in each. The cell density gradually increased by the 14th day, but the proportion of active cells declined after 7 days from 95% to 65%, probably due to the insufficient mass transfer of growth medium nutrients inside the large conglomerates.

**Figure 2:**
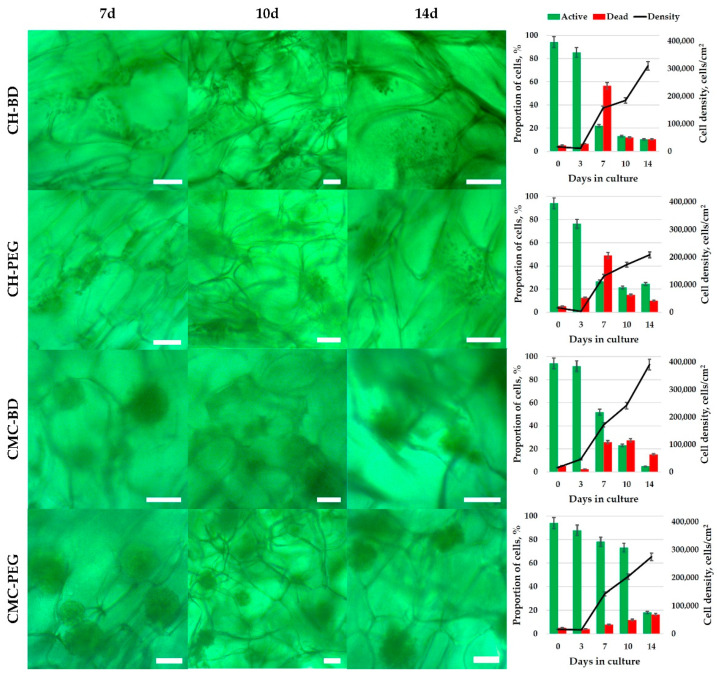
The results of microscopic observation and flow cytometrical analysis of human embryonic kidney cell line HEK-293T cultivated for 1, 2, 3, 4, 7, and 14 days in CMC- and chitosan(CH)-based cryogels cross-linked with BDDGE (CMC-BD, CH-BD) or PEGDGE (CMC-PEG, CH-PEG). The cells were stained with H_2_DCFDA to assess the mitochondrial activity, and DAPI to stain dead cells. The data are presented as a mean of three independent experiments. Standard deviations did not exceed 5%. Scale bar—100 µm.

The cells seeded into cryogels (3D conditions) passed a short-term lag phase, but had already begun to form distinct spheroids by the third day (Figure 2 and Figure S5, Supplementary Materials). At this phase, 80–90% of all cells were active. By the 7th day, the differences in cell activity and viability between CH and CMC cryogels became noticeable; the proportion of active cells decreased abruptly to 20–30% in chitosan cryogels, while in CMC cryogels it was 50–80%. It is noteworthy that a slightly higher (by 5–10%) proportion of active cells was observed in cryogels cross-linked with PEGDGE, which had higher elasticity, swelling capacity and, in case of CMC, larger pore size. Further cultivation resulted in a gradual decrease of active cells to 5–20%, but the proportion of dead cells did not exceed 20% by the 14th day, with the total cell density continuing to grow, reaching 200–300 × 10^3^ cells/cm^2^ in chitosan cryogels and 300–400 × 10^3^ cells/cm^2^ in CMC cryogels (Figure 2).

The morphology of 3D cell structures growing in cryogels was dependent on the nature of the polymer. The HEK-293T cells tended to grow in layers in chitosan cryogels, covering all inner surfaces of the pores (Figure S5, Supplementary Materials). This might be one reason for the faster reaching overgrowth phase by the 7th day and the inhibition of cell activity. Similar observations we reported for HCT 116 cells in CH-BD cryogel [31]. CMC cryogels promoted cell–cell interactions and spheroid growth due to the lack of cell–substrate interactions, which dominated in chitosan cryogels. This helped the cells to reach a higher cell density, keeping acceptable activity in CMC-based scaffolds. The activity inhibition which began after 10 days probably started when the spheroids grew too large for the nutrients to penetrate inside and be accessible for all layers of the cells.

### 2.3. Bacteria Colonization and Viability in Cryogels

Two bacterial strains, gram-positive *Staphylococcus aureus* and gram-negative *Pseudomonas* *fluorescens*, were cultured in cryogel scaffolds in DMEM media. DMEM was selected to assure the same cultivation conditions as at the co-culturing of HEK-293T cells with bacteria (Section 2.4 and Section 2.5). In preliminary experiments, bacteria growth in Nutrient Broth (NBr) and DMEM was investigated at the same inoculation dose of 10^4^ bacteria (Figure S4, Supplementary Materials) with the conclusion about DMEM suitability for both species, although the bacteria concentration was higher in NBr for *S. aureus* and in DMEM for *P. fluorescens.*

**Figure 3:**
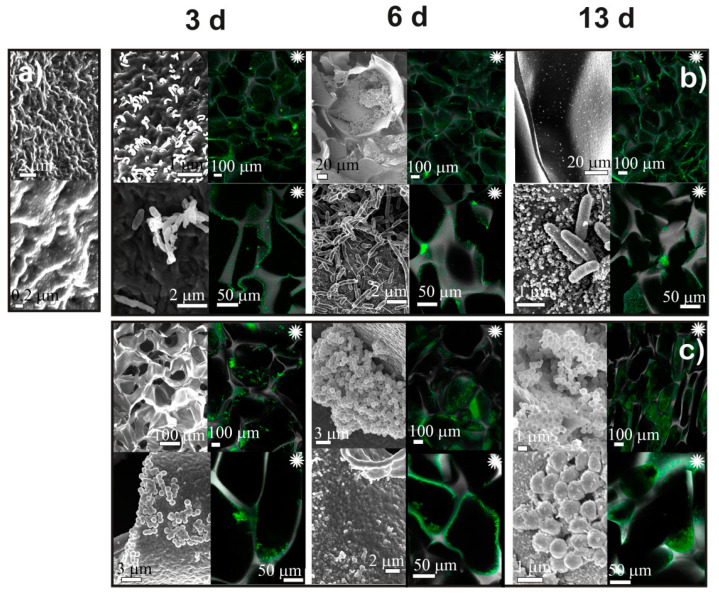
The results of SEM and CLSM observations of CH-BD cryogel before (**a**) and after cultivation for 3, 6, and 13 days *Pseudomonas fluorescens* (**b**) and *Staphylococcus aureus* (**c**). Black and white images—SEM, colored images marked with asterisk (∗)—CLSM.

**Figure 4:**
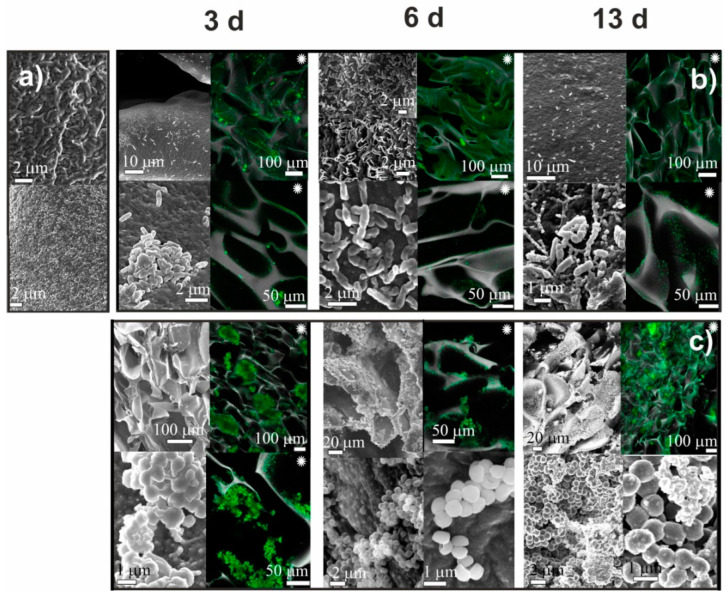
The results of SEM observation of CH-PEG cryogel before (**a**) and after cultivation for 3, 6, and 13 days *Pseudomonas fluorescens* (**b**) and *Staphylococcus aureus* (**c**). Black and white images—SEM, colored images marked with asterisk (∗)—CLSM.

After culturing bacteria for 3, 6, and 13 days, cryogels were investigated using SEM and CLSM. Figure 3 and Figure 4 show that by the third day the surfaces of the chitosan (CH-BD and CH-PEG) cryogels were predominantly populated with single *P. fluorescens* 1574 bacteria, whose deformed shape and broad size distribution indicated a depressed state of the adhered population. At the same time, surface areas covered with biofilm at different stages of maturity were clearly identified. The adhesive character of *P. fluorescens* interactions with the CH-BD and CH-PEG surfaces was also evident from CLSM images. On the sixth day of *P. fluorescens* cultivation, the pattern of surface colonization was not significantly changed on both cryogels. By the 13th day of cultivation, local aggregates of cells, microcolonies connected by extracellular polymeric substances (matrix), and areas of a mature biofilm were observed, with higher density on CH-PEG cryogel surface. Some bacteria were connected by pili, multi-subunit protein polymers, which have a pivotal role in the colonization of specific host tissues by many pathogenic bacteria [39]. Hyphae-forming cells and branched cells were clearly visible on the surface of the CH-PEG cryogel which indicates an incomplete division process, when several chromosomes remained in a single bacterium. However, the presence of large biofilm-free areas on the cryogel surfaces even after the prolonged cultivation period in the media enriched with nutrients suggests that chitosan cryogels indeed demonstrated moderate antimicrobial activity against *P. fluorescens* via preventing growth of the adhered bacteria. It is worth mentioning that the *P. fluorescens* biofilm density was higher on CH-PEG cryogel, although, taking into account that bacteria more easily colonize the harder hydrophobic surfaces, one could expect the slower growth of biofilm on the CH-PEG cryogels, which had higher swelling capacity and lower Young’s modulus in comparison with the CH-BD cryogel.

Although the same trend to denser bacterial population on CH-PEG cryogel surface was observed for *S. aureus*, in comparison with *P. fluorescens*, the growth rate of this strain was significantly higher on both chitosan cryogels. Already after 3 days of *S. aureus* cultivation, large colonies, which were more numerous in the bulk of CH-PEG cryogel, were observed by CLSM (Figure 4). Formation of a dense bacteria layer inside the cryogel pores indicated strong adhesion of *S. aureus* to the chitosan surface. On the sixth day, mature biofilms, in which deformed and melted bacteria were connected with the extracellular matrix, were observed in the SEM images of CH-BD cryogel; by the thirteenth day, biofilm mainly consisted of melted cellular debris almost completely covering the surface. Only a few separate bacteria were detected at the surface. The evolution of biofilm on CH-PEG cryogel featured a larger number of separate bacteria on the top of biofilm, the looser structure of biofilm with numerous channels, and the partial collapse of the scaffold porous structure. This suggested that the CH-PEG cryogel was degraded and consumed by *S. aureus*, possibly stimulating bacterial growth.

**Figure 5:**
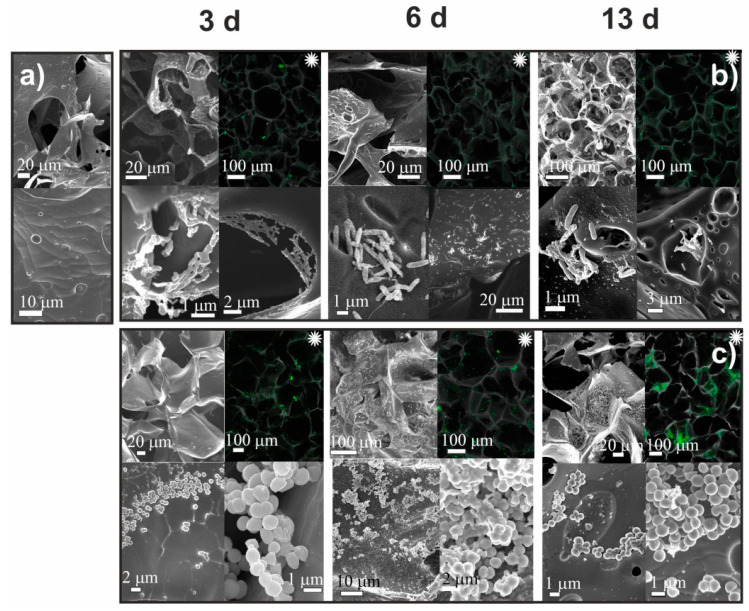
The results of SEM observation of CMC-BD cryogel before (**a**) and after cultivation for 3, 6, and 13 days *Pseudomonas fluorescens* (**b**) and *Staphylococcus aureus* (**c**). Black and white images—SEM, colored images marked with asterisk (∗)—CLSM.

**Figure 6:**
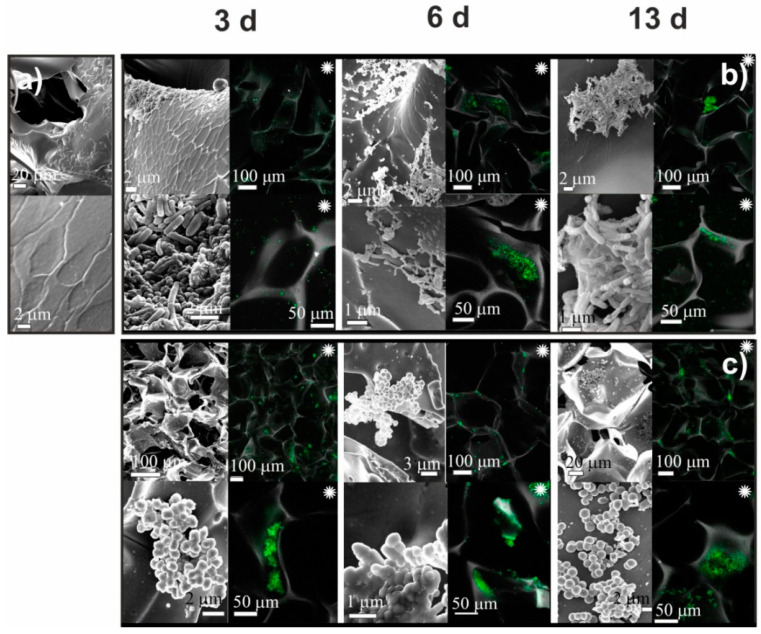
The results of SEM observation of CMC-PEG cryogel before (**a**) and after cultivation for 3, 6, and 13 days *Pseudomonas fluorescens* (**b**) and *Staphylococcus aureus* (**c**). Black and white images—SEM, colored images marked with asterisk (∗)—CLSM.

In contrast to adhesive interactions between the surface of chitosan cryogels and bacteria of both strains, CLSM images of CMC cryogels (Figure 5 and Figure 6) after *S. aureus* and *P. fluorescens* culturing showed that bacteria were mainly located inside the pores or in the “pockets” (defects of macroporous structure) and did not adhere to the cryogels surface, as was observed for chitosan cryogels. On the third day, only several deformed and fused cells of different sizes were observed on the surface of CMC cryogels. By the sixth day, mesh structures with numerous contacts between bacteria debris occupied insignificant surface area. By the 13th day, a few fragments of mature biofilm were visualized on the surface of CMC-BD and CMC-PEG cryogels. Bacteria in biofilms had normal morphology with contact pili and frothy formations at the surface.

**Figure 7:**
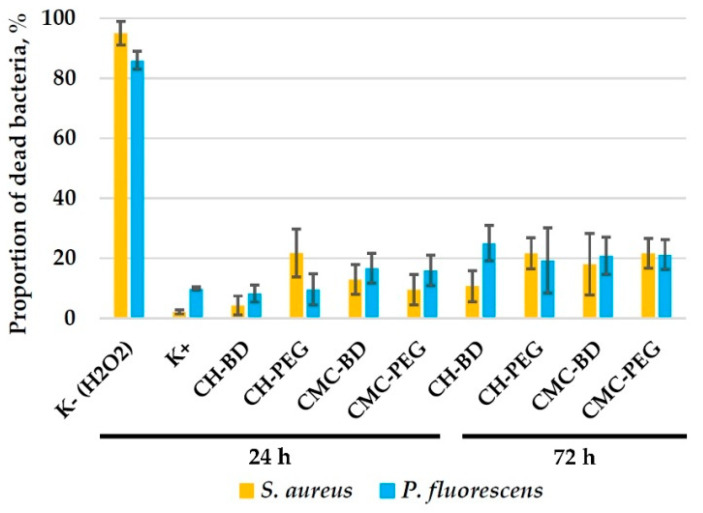
The results of flow cytometrical analysis of bacteria *P. fluorescence* and *S. aureus*. The cells were stained with SYTO™ 9 to detect all nucleic-acid-containing cells (for more precise detection of bacteria), and PI to stain dead cells. The data are presented as a mean of three independent experiments ± SD.

The proportion of dead bacteria in the control (culturing in CO_2_-incubator in adhesive plates without shaking) reached 5–10% after 24 h (Figure 7). About 85–95% of bacteria died after the incubation with 10 mM H_2_O_2_. When cultured in CMC and chitosan cryogels, the percentage of dead bacteria in suspension was 5–25%, with a slightly decreased viability after 72 h culturing compared to 24 h.

### 2.4. Co-Culturing of HEK-293T Cells with Bacteria in 2D-Conditions

Before the co-cultivation of HEK-293T cells with bacteria in 3D-matrices, it was necessary to evaluate the dynamics of changes in the functional state and number of human cells and bacteria during their co-cultivation in 2D systems (adhesive culture plates). HEK-293T cells were grown to a density of about 30–50% of the surface area of the wells, followed by the inoculation of bacteria of one of the two tested species (*P. fluorescens* or *S. aureus*).

**Figure 8:**
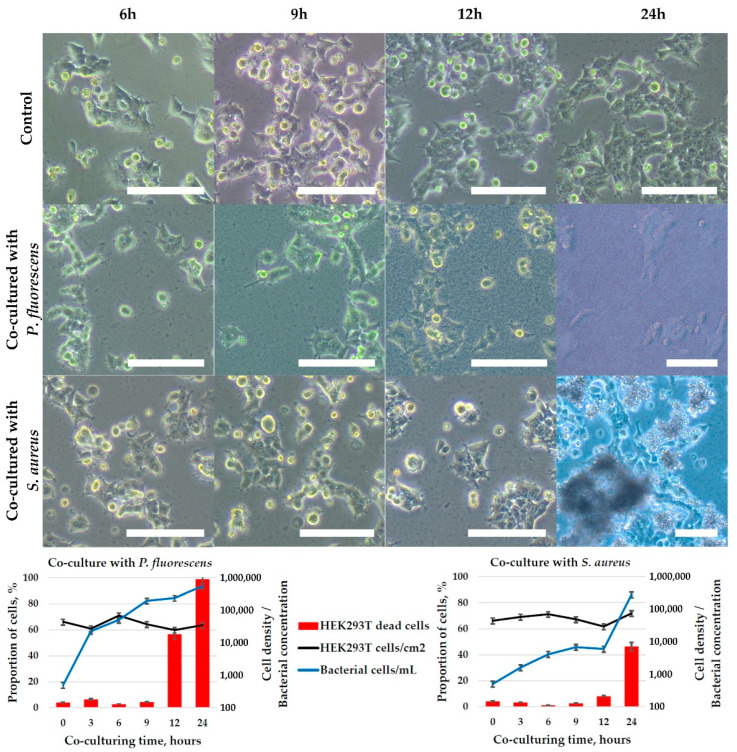
The results of microscopic observation and flow cytometrical analysis of human embryonic kidney cell line HEK-293T co-cultivated in adhesive conditions with bacteria *P. fluorescence* or *S. aureus*. The cells were stained with SYTO™ 9 to detect all nucleic acid-containing cells (for more precise detection of bacteria), and DAPI to stain dead cells. The data are presented as a mean of three independent experiments. Standard deviations did not exceed 5%. Scale bar—100 µm.

The results of co-culturing for 24 h are shown in Figure 8. *S. aureus* bacteria were characterized by slow growth during the first 12 h of co-cultivation from 500 to about 6000 bacteria/mL and a sharp logarithmic growth between 12 and 24 h to about 300 × 10^3^ bacteria/mL. *P. fluorescens* showed rapid growth from 500 bacteria/mL at the moment it was inoculated into the co-culture to 550 × 10^3^ bacteria/mL after 24 h. The presence of bacteria caused the inhibition of HEK-293T cell proliferation, however, a noticeable decrease in their density and changes in their morphology (HEK-293T cell detachment, greater spreading on the substrate, granulation of cell content, etc.) did not occur. After 12 h of co-cultivation, the toxic effect of two types of bacteria on HEK-293T cells was observed: the proportion of dead cells increased from 5–7% to 15–50%. *P. fluorescens* had a higher toxic effect, which is probably directly related to the higher growth dynamics of this bacterial species and the higher concentration of bacteria at the time of 12 h of co-cultivation, compared to *S. aureus*. After 24 h of co-cultivation with *P. fluorescens*, the viability of HEK-293T cells decreased to 2–5%. In the presence of *S. aureus*, the decrease of HEK-293T cells viability was much lower and reached 50% after 24 h of co-culturing. 

### 2.5. Co-Culturing of HEK-293T Cells with Bacteria in 3D Conditions

**Figure 9:**
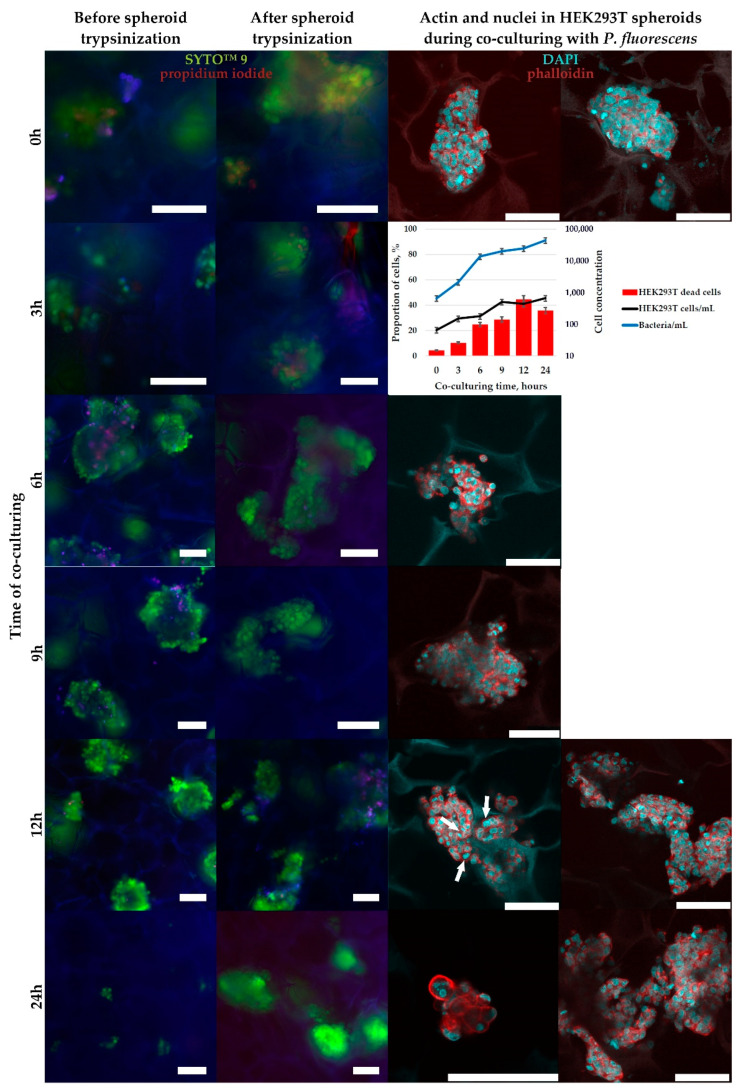
The results of microscopic observation and flow cytometrical analysis of HEK-293T cells co-cultivated in 3D CMC-BD cryogel with bacteria *P. fluorescence*. For trypsinization tests the cells were stained with SYTO™ 9 detect all nucleic acid-containing cells (for more precise detection of bacteria) and propidium iodide (to stain dead cells). For cytoskeleton visualization the cells were stained with phalloidin (actin staining) and DAPI (nuclei staining). Cells with mitotic spindle are marked with arrows. For flow cytometrical analysis the cells were stained with SYTO™ 9 to, and DAPI to stain dead cells. The data are presented as a mean of three independent experiments. Standard deviations did not exceed 5%. Scale bar—100 µm.

**Figure 10:**
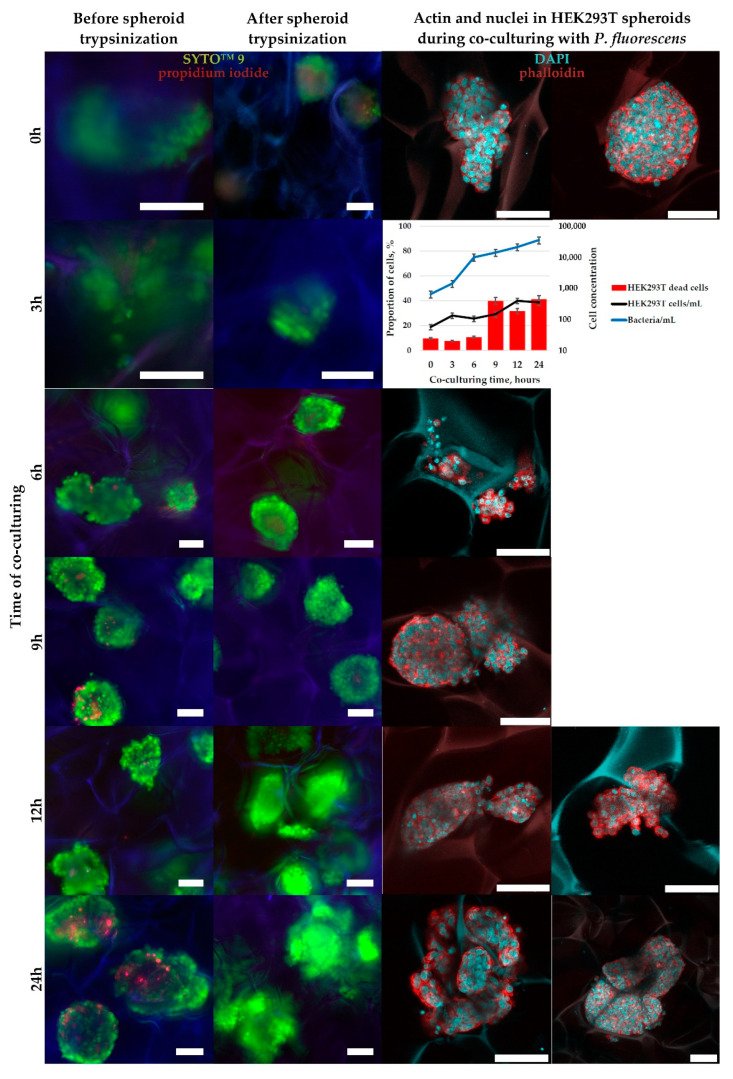
The results of microscopic observation and flow cytometrical analysis of HEK-293T cells co-cultivated in 3D CMC-cryogels cross-linked with PEGDGE with bacteria *P. fluorescence*. For trypsinization tests the cells were stained with SYTO™ 9 detect all nucleic acid-containing cells (for more precise detection of bacteria) and propidium iodide (to stain dead cells). For cytoskeleton visualization the cells were stained with phalloidin (actin staining) and DAPI (nuclei staining). For flow cytometrical analysis the cells were stained with SYTO™ 9 to, and DAPI to stain dead cells. The data are presented as a mean of three independent experiments. Standard deviations did not exceed 5%. Scale bar—100 µm.

HEK-293T cells, preliminarily grown in CMC-BD (Figure 9) and CMC-PEG (Figure 10) cryogels for 10 days to form spheroids with a size > 100 µm, were co-cultured with *P. fluorescens* for 24 h. After the inoculation of bacteria into cryogels, the changes in the bacterial growth, the number of HEK-293T cells, and their viability were monitored. The dynamics of bacterial division in cryogels was comparable to that during co-cultivation in adhesive 2D conditions (Figure 8), but the toxic effects from the bacteria on HEK-293T cells was lower in 3D conditions; from flow cytometrical data, the maximal level of dead human cells (about 40%) was observed after 12–24 h of co-culturing (Figure 9 and Figure 10), while in 2D conditions it reached 50–100% at this time points (Figure 8).

It should be mentioned that trypsinization, applied for the dissociation of HEK-293T spheroids to perform flow cytometrical analysis, was incomplete, as demonstrated by SYTO™ 9 with propidium iodide staining (Figure 8 and Figure 9). This was not only the complication of the current study protocols or cell line used, but was also reported previously by other researchers [40,41]. In our case, HEK-293T cultivated in DMEM with 10% FBS may have bound many proteins from the medium, forming a kind of coating around cells, which protects contact proteins between cells from the action of trypsin. The impossibility of full spheroid dissociation might be a reason for the observed discrepancy in microscopic (approximate number of PI-positive dead cells is low) and flow cytometrical data (the proportion of DAPI-positive dead cells reached 40%), because dead cells, which were mostly located at the spheroid’s surface, were more easily trypsinized and washed out for flow cytometry analysis than live cells with stronger cell-to-cell contacts. Hence, the relative content of dead cells in trypsinized cell suspension was higher than visualized under the microscope in intact spheroids.

## 3. Discussion

### 3.1. Cryogels Fabrication and Characterization

Considering that softer CMC-cryogels provided microenvironment-stimulating HCT 116 cell–cell interactions in contrast to stiffer chitosan cryogels with strong cell–substrate interactions, we were motivated to further explore how surface chemistry, porosity, and mechanical properties affect the morphology of multicellular aggregates of another adherent cell line (HEK-293T), and bacteria adhesion and growth in CMC and chitosan cryogels. The obtained information can be of interest for the targeted choice of the scaffolds: (i) to mimic specific biological environment in 3D-tissue models; (ii) to provide dual antimicrobial and regenerative properties for degradable bioimplants; (iii) to develop 3D mammalian cell-bacteria models to investigate host–pathogen interaction mechanisms and test new antimicrobial strategies.

The key point in the structural difference between chitosan and CMC is the presence of carboxylic groups in CMC, which determines high hydrophilicity, good solubility in neutral and alkaline media, and the negative charge of this derivative at physiological pH in contrast to acid-soluble and positively charged chitosan. Aside from the earlier reported strong dependence of cell adhesion and proliferation on the chitosan films on the number of amino groups at the surface [42,43], the difference in solubility and types of functional groups in CMC and chitosan significantly affects the selection of the cross-linking agents and the pH of the media for hydrogel and cryogel fabrication. At the same time, the cross-linking mechanism and density affect porosity, swelling behavior, mechanical properties and, thus, the performance of cryogels as biomaterials [44].

Chitosan-based chemically cross-linked scaffolds are usually obtained in acidic media using dialdehydes, mostly glutaraldehyde (GA) [45,46], whose cytotoxicity still has to be compromised [24]. FDA-approved for biomedical application diglycidyl ethers of glycols (DGE) [47], which can cross-link biopolymers via amino-, hydroxyl-, or carboxyl groups at pH > 10 [48,49,50], were earlier used for CMC and other water-soluble chitosan derivatives [10,37]. Due to the insolubility of chitosan in alkaline media, this type of cross-linker was known only for the post-modification of the material, whose porosity was generated by phase separation and freeze-drying [51]. However, in comparison with cross-linking in partially frozen polymer solution, this approach does not allow for the broad variation of the scaffold parameters.

Only recently, we have demonstrated that the reaction between DGE and chitosan can proceed in HCl media at pH ~5 at subzero temperature and yield cryogels with mechanical properties and permeability dependent on the cross-linking density and type of cross-linker used [33]. The biocompatibility of the obtained chitosan-based scaffolds was confirmed in a mouse model; a moderate inflammatory reaction around the implants was accompanied by formation of a normal granulation tissue, and no toxic, immunosuppressive, and sensitizing effects on the recipient’s tissues have been observed [33].

In addition to BDDGE used as a cross-linker in our previous study on HCT 116 3D-culturing in CMC and chitosan cryogels [31], we have selected PEGDGE with Mn~500 for the following reasons: (i) although BDDGE was more reactive, PEGDGE was a more efficient cross-linker for polyethyleneimine due to the lower probability of undesired side-chain grafts formation [52]; (ii) long, flexible PEG chains can improve the elasticity and robustness of chitosan cryogels, which are intrinsically more brittle in comparison with CMC-cryogels; (iii) PEG-containing materials are well known to reduce bacterial adhesion [10,53].

The comparative analysis of CMC and chitosan cryogels characteristics (Figure 1) showed that all materials had macroporous structure with pore size > 150 µm, swelling capacity from 4000 to 6500% depending on cross-linking conditions, good mechanical properties with compressive strength ~11 kPa and ~6 kPa at 75% strain for chitosan and CMC, respectively. All cryogels sustained uniaxial deformation at least up to 80% of their original height, and returned to their original size and shape after stress unloading.

Using PEGDGE as a cross-linker for chitosan allowed the significant improvement of cryogel elasticity (lower hysteresis in stress loading/unloading cycle) and swelling capacity without changes of the pore size. CMC-PEG cryogel, in comparison with CMC-BD, had larger pore size, higher swelling capacity and higher Young’s modulus, that can be related to the higher cross-linking efficiency of PEGDGE in comparison with BDDGE under the selected conditions. Poor control of CMC-hydrogels composition by the amount of PEGDGE, when cross-linking was performed at 60 °C [37], suggested that the undesired grafting of PEGDGE to the side chain instead of cross-links formation was a typical problem of hydrogels fabrication using this type of cross-linkers. This effect was less pronounced, when cross-linking was performed at subzero temperature. 

To summarize, the selection of CMC and chitosan as polymers, and BDDGE and PEGDGE as cross-linkers allowed fabrication of cryogels with comparable pore sizes and Young’s moduli but different surface chemistry (CH-PEG and CMC-PEG); similar surface chemistry and mechanical properties but different pore sizes (CMC-BD and CMC-PEG); similar pore sizes and surface chemistry but different mechanical properties (CH-PEG and CH-BD).

It is worth mentioning that all fabricated cryogels were permeable for the fluids and, thus, can be applied in flow-through systems for human cells and bacteria culturing and co-culturing. Taking into account that the fluid flow deeply affects bacterial transport and colonization on the surfaces with still poor understanding mechanism of this phenomena [54,55], cryogels can be an alternative to the existing models to study bacteria colonization on bioimplant surface and cell–microorganisms interactions under dynamic conditions in 3D more relevant to the biological systems than static experiments in wells.

### 3.2. Culturing HEK-29 Cells in 2D and 3D

The tendency of cells to interact with each other or with a substrate (matrix) influences the cell growth pattern in culture. If cells do not find specific substrate properties (stiffness, surface charge, chemical groups or proteins, etc.) and do not significantly inhibit each other proliferation by contact, they can grow in all three dimensions forming spheroids. Such pattern was observed in our previous study with HCT 116 cells cultivated in CMC cryogels with Young’s moduli 3–13 kPa, in which the adhesion of cancer cells to each other prevailed over the adhesion to the surface [31]. Here using another adherent cell line HEK-293T we have proved validity of the hypothesis that nature of polymer, i.e., surface chemistry, was very important to guide morphology of 3D cellular structures in chitosan and CMC-based scaffolds. Numerous HEK-293T spheroids with the size above 100 µm were observed in both CMC-BD and CMC-PEG cryogels by the 7th day of cultivation (Figure 2). The advantage of CMC-PEG cryogel for HEK-293T culturing most likely results from notably larger average pore size (232 µm) in comparison with CH-BD cryogel (156 µm). This is in line with a decrease in HCT 116 spheroid size in CMC cryogels cross-linked with BDDGE at different densities [31].

The growth of cells is limited by medium nutrients availability for layers of cells from the surface to the inner part of spheroids [56]. If the substrate meets the requirements of the cells, the cells will tend to adhere to the substrate rather than to each other. This pattern was observed during cultivation of HCT 116 cells in stiff chitosan-cryogels (Young’s modulus 41 kPa) [31]. Despite the phase-contrast microscopy did not show almost no distinction in the morphology of HEK-293T multicellular structures formed in CH-BD and CH-PEG cryogels (Figure 2), confocal microscopy revealed some difference in the cell growth in cryogels formed from the same polymer, but cross-linked either BD or PEG (Figure S5, Supplementary Materials): the cell-substrate interactions were stronger in BDDGE cross-linked cryogels. The higher Young’s modulus and lower swelling capacity of CH-BD cryogel (Figure 1) promoted cell-substrate interactions and cell spreading with flattening of the cellular shape.

The pore sizes in chitosan cryogel were >200 µm, but the cell growth was mostly limited by the available surface area; and when overgrowth happen, the contact inhibition resulted in degradation of culture that is more critical for mortal cell cultures. If cells cannot find necessary substrate and experience high contact inhibition, they will not proliferate and will die. This explains lower viability of cells in chitosan cryogels at prolonged culturing time.

### 3.3. Bacteria Adhesion and Biofilm Formation on Chitosan and CMC Cryogels

As it was mentioned above, investigation of microorganism’s interactions with scaffolds for cell culturing and tissue engineering is crucial for understanding risks of post-surgical complications and bioimplant failure due to adhesion and colonization of bacteria, which can contaminate implant surface despite careful sterile and hygienic surgical conditions during implantation [57]. To address this issue, an ideal implanted material should have the dual functions at the same time: preventing bacterial infection and stimulating tissue regeneration [2]. In general, bacterial biofilm formation can be prevented on materials, which have either antifouling properties, i.e., are characterized by the low adhesion of bacteria, or antimicrobial properties and the ability to inhibit the growth or kill bacteria approaching or adhered to the surface [58].

Since positively charged materials stimulate bacterial adhesion, but prevent growth of adhered bacteria [53], we have expected that chitosan cryogels with a low positive surface charge at physiological pH will resist biofilm formation due to this mechanism of action. CMC-cryogels, as very hydrophilic and negatively charged at physiological pH, are, in contrast, were expected to demonstrate antifouling behavior.

Literature data on antimicrobial properties of chitosan and CMC-based materials, including cryogels, are very contradictory that can result from different evaluation methods. In most cases, when high antimicrobial activity was observed, agar tests or shake flask test were used to make a conclusion, as, for example was carried out in [10,59] for activity against *S. aureus*. At the same time, testing chitosan cryogels with and without polylysine against gram-positive methicillin-resistant *S. aureus (MRSA)* and gram-negative *E. coli* showed no significant antibacterial effect of unmodified chitosan cryogel [5].

Our results on adhesion of *P. fluorescence* and *S. aureus* on CMC and chitosan cryogels (Figure 3, Figure 4, Figure 5 and Figure 6) have shown that bacteria strongly adhered to the surface of chitosan cryogels forming fragments of biofilm by the 3rd day of culturing. Surprisingly, that the highest rate of colonization by both strains was observed on CH-PEG cryogel (Figure 4). At the late stages of observation (13th day) this cryogel was even partially degraded by *S. aureus.* Changes in bacteria morphology and the presence of large biofilm-free areas on the cryogel surfaces even after the prolonged cultivation period in the media enriched with nutrients suggests that chitosan cryogels indeed demonstrated moderate antimicrobial activity against *P. fluorescens* via preventing growth of the adhered bacteria, as could be expected from the low positive surface charge of these cryogels. However, antimicrobial activity against *S. aureus* was low and large surface areas were covered with matured biofilm. Figure 7 demonstrated that viability of bacteria in suspensions in contact with chitosan-based cryogels was in most cases above 80%.

CMC-cryogels demonstrated behavior characteristic for antifouling materials (Figure 5 and Figure 6). At 3rd day of culturing, CLSM images show that bacteria are mostly trapped in cryogels pores and not adhered to the surface in contrast to strong adhesion on chitosan-based cryogels. Loose microcolonies of bacteria were observed in both CMC-BD and CMC-PEG cryogels, but the occupied surface area was insignificant in comparison with CH-BD and CH-PEG cryogels. Bacteria in fragment of biofilms on CMC-cryogels mostly had normal morphology with contact pili and frothy formations at the surface. This confirms the initial hypothesis that CMC-cryogel does not kill bacteria but prevent biofilm formation due to the antifouling properties determined by high hydrophilicity and electrostatic repulsion between bacteria and negatively charged scaffold surface.

### 3.4. Bacteria and HEK-293T Cells Co-Culturing in 2D- and 3D-Conditions

Co-culturing in 2D-conditions of human embryonic kidney cell line HEK-293T with two species of bacteria have shown the rapid development of bacterial infection in vitro, with ~50% and 100% cell culture degradation within 24 h in the presence of *S. aureus* and *P. fluorescens*, respectively (Figure 8). This is not surprising due to facts that bacteria of both tested species grow logarithmically reaching stationary phase during 10–15 h (Figure S6, Supplementary Materials) and produce potentially toxic compounds. The human cells were in contact with many thousand bacteria and continuous mixing by Brownian motion of medium with toxins resulted in the high susceptibility of cells to the presence of bacteria toxins.

Although *S. aureus* had higher affinity to the cryogels surface, to co-culture HEK-293T cells and bacteria in 3D we have chosen *P. fluorescens*, as its presence induced higher damage for human cells in 2D culture. To obtain more contrast and reliable results for a short period of co-culturing, we decided to inoculate *P. fluorescens* to HEK-293T spheroids in CMC cryogels, in which cells had the higher activity and viability by the 10th day of cultivation in comparison with cultivation in chitosan-based cryogels. Additionally, CMC cryogels, as low adhesive substrates for bacteria, are more suitable for long-term experiments and can be used as flow-type alternative to the mammalian cell-bacteria co-culturing models in the wells with separating membrane.

When co-cultured with *P. fluorescens* in 3D-conditions using CMC-BD and CMC-PEG cryogels, at least 50% of HEK-293T cells retained viability after 24 h (Figure 9 and Figure 10). Furthermore, microscopic observations allowed assumption that actual HEK-293T cells viability was even higher than calculated from the flow cytometry analysis. To assess the influence of bacterial presence in co-culture on the cytoskeleton of HEK-293T cells we used phalloidin staining of cellular actin and DAPI for visualization of cell nuclei (Figure 9 and Figure 10). Actin is localized in cells as an extensive network of filaments. Most nuclei had round shapes. After 12 h of co-culturing with *P. fluorescens* the HEK-293T cells with a mitotic spindle were still visualized (Figure 9), pointing to the low influence of bacteria presence on HEK-293T cells proliferation at this time point. The number of visible nuclei disturbances (segmentation or shape changing) increased by 24 h, without a noticeable increase in the number of cells, indicating an extensive inhibition of HEK-293T cell growth.

The reduced toxic effect of bacteria on HEK-293T cells in 3D culture can be related to the protection of the cells inside spheroids by the external cell layer and other factors, which determine different sensitivity of cells to drugs in 2D and 3D. Such co-culture model can be useful for investigation of nephrotoxicity of antimicrobial strategies.

## 4. Materials and Methods

### 4.1. Materials

Low molecular weight (30 kDa) chitosan (CH) and N,O-(carboxymethyl)chitosan (CMC) were purchased from BioLog Heppe GmbH (Landsberg, Germany). The chitosan degree of acetylation (DA = 0.9), CMC degree of carboxyalkyl substitution (DS = 1.49), and monomer composition of CMC were determined by ^1^H NMR spectroscopy (Figure S1 and Table S1, Supplementary Materials). The cross-linking agents—1,4-butanediol diglycidyl ether (BDDGE) and poly(ethylene glycol) diglycidyl ether with average Mn 500 (PEGDGE)—were purchased from Sigma-Aldrich (St. Louis, MO, USA).

### 4.2. Fabrication of Cryogels

The 3% chitosan solution was prepared by the dissolution of the polymer powder in hydrochloric acid at an equimolar NH_2_:HCl ratio; then, pH was adjusted to 5 with 0.1 M NaOH solution. A CMC solution of the same concentration was prepared in distilled water, and the resulting pH of the solution was 10.2. The calculated amounts of the cross-linkers, corresponding to the molar ratios BDDGE:CH 1:4, BDDGE:CMC 1:2, PEGDGE:CH 1:12, PEGDGE:CMC 1:8, were added dropwise under constant stirring to the polymer solutions to obtain cryogels CH-BD, CMC-BD, CH-PEG, and CMC-PEG, respectively (formulations for cryogels fabrication are summarized in Table S2, Supplementary Materials). Then, the solutions were immediately placed into plastic syringes with an inner diameter of 10 mm and kept in a freezer (Liebherr, Kirchdorf an der Iller, Germany) at −10 °C for 12 or 7 days for chitosan or CMC, respectively. After thawing, the cryogels were washed with distilled water using a peristaltic pump (Ismatec, Wertheim, Germany) to remove unreacted chemicals.

### 4.3. Characterization of Cryogels

The swelling of the cryogels was determined from the difference in weights of the swollen and dry material (the measurements were performed for freshly prepared cryogels from wet to dry state). To determine the contribution of water absorbed in macropores to the total swelling, the swollen cryogels were first squeezed by fingers and weighted.

The morphology of the cryogels stained with fluorescein (chitosan cryogels) or rhodamine (CMC cryogels) was analyzed using confocal laser scanning microscopy (CLSM), as described in Section 4.8.

Fourier transform infrared (FT-IR) spectra were recorded using an IR Affinity-1 spectrometer with a MIRacle 10 FTIR accessory (Shimadzu, Kyoto, Japan).

Uniaxial mechanical tests at a constant speed of 0.01 mm/s were performed for the swollen cylindrically shaped cryogels with a diameter of 10–15 mm and height of 8–10 mm using a Physica MCR 301 rheometer (Anton Paar GmbH, Graz, Austria). Loading and unloading strain–stress curves have been calculated from the measured normal force (F_N_) and gap. In selected experiments several loading–unloading cycles have been recorded for the cryogels placed in a Petri dish filled with water up to 10% of the cryogel height.

The Young′s modulus (E) was calculated from the linear region (up to 14% strain) of the loading curve:

E=l0·FNS·Δl

where F_N_ is the normal force (N), l_0_ is the initial sample height (m), Δl is the change in the sample height (m), and S is the area of the material (m^2^).

### 4.4. HEK-293T Cell Cultivation

The 24-well culture plates (TPP, Trasadingen, Switzerland), ultra-low attachment plates (Corning^®^ Costar^®^, Somerville, MA, USA), and cryogel disks were used to grow HEK-293T cells (ab255449, Abcam, Cambridge, UK) in adhesive, ultra-low attachment, and 3D conditions, respectively.

HEK-293T cells were seeded in adhesive and ultra-low attachment plates at a density 10^4^ cells/well in 1 mL of Dulbecco’s modified Eagle’s medium (DMEM, #12800017, Gibco™, Thermo Fisher Scientific, Altrincham, UK) supplemented with 10% (*v*/*v*) fetal bovine serum (FBS, HyClone, Logan, UT, USA), 3.7 mg/mL sodium bicarbonate (Sigma-Aldrich), 1× mixture of non-essential amino acids (MEM NEAA, Waltham, MA, USA, Gibco), 100 U/mL penicillin (Gibco), and 100 µg/mL streptomycin (Gibco).

The fabricated cryogels were cut into disks (diameter of 10 mm, thickness of 4 mm). Each disk was placed in a well of the 24-well TPP culture plate, consistently washed with 5 mL of Dulbecco’s phosphate buffer saline (DPBS, Sigma-Aldrich) without Ca^2+^ and Mg^2+^, and 5 mL of DMEM with additives. Disks were incubated with DMEM at +37 °C, 5% CO_2_ for 2 h for equilibration, and washed with 1 mL of DMEM with additives again. All liquid was then squeezed from the cryogel. HEK-293T cells were seeded on the top of a cryogel disk at a density of 10^4^ cells/cryogel in 80 µL of DMEM, with additives to fill up all cryogel volume. Then, 1 mL of DMEM with additives was carefully appended to the well outside the cryogel disk.

All samples were cultivated at +37 °C, 5% CO_2_ and 90% relative humidity. The medium was changed every 2 days. The cells were monitored daily under a CKX41 inverted microscope (Olympus, Shinjuku City, Tokyo, Japan) equipped with phase-contrast optics and imaged with an Axiocam 105 color digital camera (Carl Zeiss, Oberkochen, Germany) in ZEN 2 (blue edition, Carl Zeiss). The viability and functional activity of cells were analyzed after 1, 2, 3, 7, and 14 days of cultivation in cryogels, as described in Section 4.7.

### 4.5. Bacterial Culturing

#### 4.5.1. Preparation of Bacterial Stocks

The bacterial strains of *Staphylococcus aureus* (21027™, ATCC, Manassas, VA, USA) and *Pseudomonas fluorescens* 1574 from the collection of the Museum of Marine Heterotrophic Bacteria (A.V. Zhirmunsky National Scientific Center of Marine Biology FEB RAS, Vladivostok, Russia) were grown overnight in Luria agar, which was obtained by adding 12 g/L Agar-Agar (Merck, Darmstadt, Germany) to LB Broth (Miller) (Sigma-Aldrich). To prepare cell suspensions, the overnight cultures were diluted with DPBS to a concentration corresponding to OD 600 1.0. Lower concentrations were obtained by serial dilution.

The precise concentration of bacteria in suspension was determined just before inoculation into cryogels, according to Section 4.7.

#### 4.5.2. Cultivation of Bacteria in Cryogels

The fabricated cryogels were washed, equilibrated with DMEM (with additives, but without penicillin and streptomycin), and squeezed, as described in Section 4.4. Bacteria were inoculated into the cryogel disks at the quantity of 500 bacteria/disk in 80 µL of DMEM with additives, but without antibiotics, to fill up all cryogel volume. Then, 1 mL of this DMEM was carefully appended to the well outside the cryogel disk. Bacteria were cultivated at +37 °C, 5% CO_2_ and 90% relative humidity. The medium was changed every 2 days. After 3, 6, and 13 days in culture, the cryogel disks with bacteria were fixed for analysis by scanning electron microscopy (SEM) and confocal laser scanning microscopy (CLSM).

The samples for SEM were fixed with 2.5% glutaraldehyde (EMS, Hatfield, PA, USA) in 0.1 M cacodylate buffer (Sigma-Aldrich, St. Louis, MO, USA), passed through the increasing concentrations of ethanol (30, 50, 70%) with 30 min incubation at each stage, dried at the critical point, and coated with chromium, then analyzed using the scanning electron microscope SIGMA VP (Carl Zeiss, Jena, Germany. The disks for CLSM were fixed as described in Section 4.8.

### 4.6. Co-Culturing of HEK-293T with Bacteria

The co-culturing of HEK-293T cells with bacteria was conducted in adhesive plates or in fabricated cryogels. The bacterial strains were grown and the concentration of bacteria in suspensions was determined immediately before inoculation into cryogels with HEK-293T, as described in Section 4.5.1.

For tests in 2D culture conditions, HEK-293T were seeded in adhesive TPP plates as described in Section 4.4 and cultivated for 2 days in the medium without antibiotics. The medium was aspirated from the wells and bacteria were inoculated at the quantity of 500 bacteria/well in 1 mL of DMEM with additives, but without antibiotics. HEK-293T cells were co-cultured with bacteria at +37 °C, 5% CO_2,_ and 90% relative humidity. After 3, 6, 9, 12 and 24 h the co-cultures were monitored under a CKX41 inverted microscope equipped with phase-contrast optics, imaged with an Axiocam 105 color digital camera, trypsinized from the wells, stained with fluorescent dyes (as described in Section 4.7), and analyzed by flow cytometry.

For the tests in 3D conditions, HEK-293T were seeded in equilibrated with DMEM cryogel disks, as described in Section 4.4, and grown for 10 days in the medium without antibiotics being changed every 2 days until visual three-dimensional cellular spheroid formation. About 30% of the medium was carefully squeezed from the cryogel (without visual disturbing the spheroids), and bacteria were inoculated into the cryogel disks at the quantity of 500 bacteria/disk in 80 µL of DMEM with additives, but without antibiotics. Then, 1 mL of this DMEM was carefully appended to the well outside the cryogel disk. HEK-293T cells were co-cultured with bacteria at +37 °C, 5% CO_2_ and 90% relative humidity. After 3, 6, 9, 12, and 24 h the co-cultures in the cryogel disks were transferred into a new well, stained with fluorescent dyes (as described in Section 4.7), imaged under inverted fluorescent microscope Axiovert 200M (Carl Zeiss), trypsinized from the disks, imaged again under the fluorescent microscope, and analyzed by flow cytometry. Another parallel of the cryogel disks with the co-cultures were fixed for CLSM at the same time points and washed as described in Section 4.8.

### 4.7. HEK-293T and Bacteria Staining and Flow Cytometry

The single cultures of HEK-293T cells (without bacteria) grown in adhesive, ultra-low attachment plates, and cryogels were washed with 1 mL of DPBS, detached with 1 mL of 0.05% (*w*/*v*) trypsin—0.02% (*w*/*v*) EDTA solution (Sigma-Aldrich) from the wells or cryogel disks, and centrifuged at 500× *g* for 5 min. A pellet of trypsinized cells from a single well of a 24-well plate or cryogel disk was re-suspended in 100 µL of DPBS with 10 µM 2′,7′-dichlorodihydrofluorescein diacetate (H_2_DCFDA) (Sigma-Aldrich) to assess the mitochondrial activity, and 1 µg/mL 4′,6′-diamidino-2-phenylindole (DAPI, Sigma-Aldrich) to stain dead cells. The cell suspension was incubated in the dark at room temperature for 10 min and then diluted with 150 µL of DPBS before analysis with a CytoFLEX flow cytometer (Beckman-Coulter, Brea, CA, USA) connected to a computer running CytExpert software (Version 2.4, Beckman-Coulter). The detailed description of flow cytometrical analysis is given in [60].

To precisely determine the concentration of bacteria in suspension immediately before inoculation into wells or cryogels, a 100 µL aliquot of bacterial suspension in DPBS was stained with fluorescent nucleic dye SYTO™ 9 (Thermo Fisher Scientific) at the final concentration of 2 µM for 10 min at room temperature and analyzed using a CytoFLEX flow cytometer (Beckman-Coulter, USA) connected to a computer running CytExpert software (version 2.4, Beckman-Coulter).

The co-cultures of HEK-293T cells and bacteria were trypsinized from the wells or cryogel disks, stained with 1 µg/mL DAPI (to detect dead HEK-293T cells) and 2 µM SYTO™ 9 (to precisely quantify bacterial concentration) in the dark at room temperature for 10 min, and analyzed by flow cytometry. The detailed description of flow cytometrical analysis is given in [60].

### 4.8. Confocal Laser Scanning Microscopy (CLSM)

Morphology of the swollen (never dried) chitosan cryogels stained with fluorescein and CMC cryogels stained with rhodamine was investigated using a Carl Zeiss LSM 800 confocal laser scanning microscope (Germany) with regular 10× and 20× objective lens. The excitation and emission wavelengths used were set at 488 and 530 nm for fluorescein and at 561 and 572 nm for rhodamine, respectively. Images were generated by optical sectioning in the xy planes along the *z*-axis with 30 optical sections with 2.5 μm intervals. The optical-section series were projected as single images and exported as TIFF files. The pore size distributions were calculated using the ImageJ software [61].

The disks for CLSM were fixed with 4% paraformaldehyde (PFA, Sigma-Aldrich) in DPBS for 30 min at +4 °C and washed three times with cold DPBS. To detect bacteria, cryogel disks were incubated with 2 µM SYTO™ 9 stain solution. To detect filamentous actin, cryogels with co-cultures were incubated overnight in a solution of Phalloidin CruzFluor™ 488 Conjugate (Santa Cruz Biotechnology, Santa Cruz, CA, USA) at +4 °C and washed three times with cold DPBS. Then the samples were stained with 10 μg/mL DAPI, Sigma in DPBS to reveal the nuclei. The stained material was stored in DPBS with the added preservative ProClin™ (Sigma-Aldrich) in the dark at +4 °C. Immediately before the microscopy analysis, the cryogel disks were placed in Vectashield^®^ antifade mounting medium (Vector Laboratories, Burlingame, CA, USA) in a Petri dish (Nunc™, Rochester, NY, USA) with a thin (0.17 mm) glass bottom. Imaging was performed using confocal microscope LSM 800 (Carl Zeiss, Oberkochen, Germany) under objectives EC Plan-Neofluar 10×/0.30 and Plan-Apochromat 20×/0.8 at excitation wavelength 488 nm and emission range 505–700 nm for SYTO™ 9, and at excitation wavelength 405 nm and emission range 400–505 nm for cryogel’s autofluorescence. The images were pseudocolored and merged using the ImageJ software, version 1.53c (NIH, Bethesda, MD, USA).

## Data Availability

Data available from the authors upon request.

## Supplementary Materials

The supporting information can be downloaded at: https://www.mdpi.com/article/10.3390/ijms232012276/s1.
