# Supplementary material for: Chitosan versus Carboxymethyl Chitosan Cryogels: Bacterial Colonization, Human Embryonic Kidney 293T Cell Culturing and Co-Culturing
Source: Int J Mol Sci. 2022 Oct 14;23(20):12276. doi: 10.3390/ijms232012276 (PMC9602999; doi:10.3390/ijms232012276)
Supplement: Supplementary file 1 [file ijms-23-12276-s001.zip › ijms-1916983-supplementary.pdf]

Supplementary materials

Chitosan versus Carboxymethyl Chitosan Cryogels: Bacterial Colonization, Human Embryonic Kidney 293T Cell Culturing and Co-Culturing

Andrey Boroda [1], Yuliya Privar [2], Mariya Maiorova [1], Irina Beleneva [1], Marina Eliseikina[1], Anna Skatova [2], Dmitry Marinin[2] and Svetlana Bratskaya [2,*]

[1] A.V. Zhirmunsky National Scientific Center of Marine Biology, Far Eastern Branch of Russian Academy of Sciences, 17, Palchevskogo street, 690041 Vladivostok, Russia

[2] Institute of Chemistry Far Eastern Branch of the Russian Academy of Sciences, 159, prosp.100-letiya Vladivostoka, 690022 Vladivostok, Russia

* Correspondence: sbratska@ich.dvo.ru

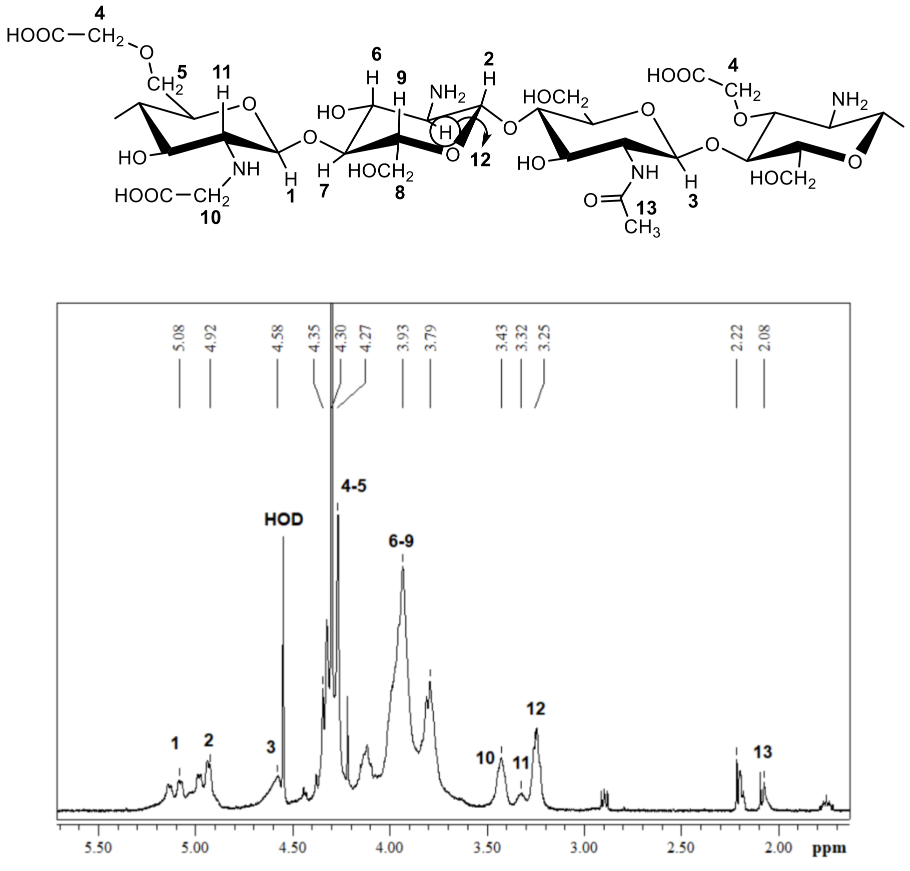

Figure S1. 400 MHz ^{1}H NMR spectrum of N,O-(carboxymethyl)chitosan

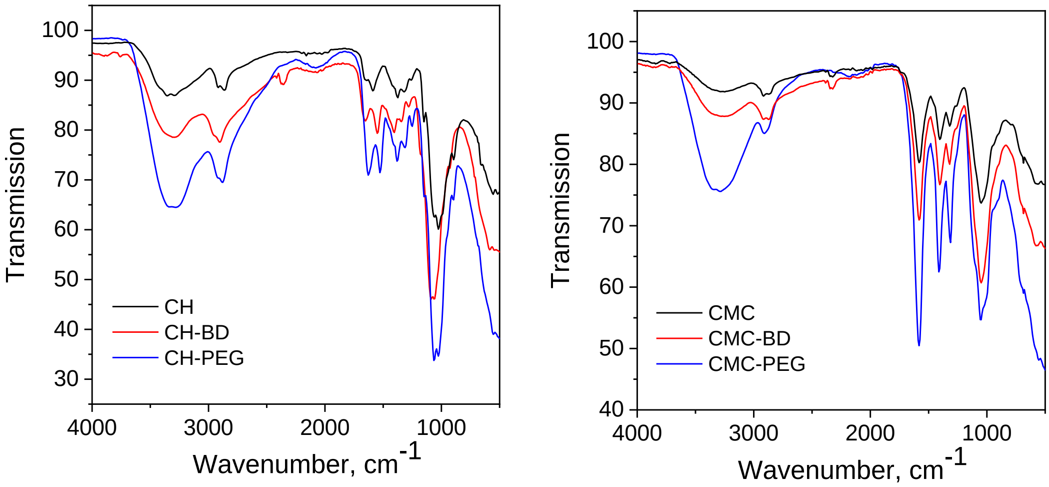

(a) **(b)**

Figure S2. FI-IR spectra: l chitosan (CH) and chitosan-based cryogels (a); CMC and CMC-based cryogels (b).

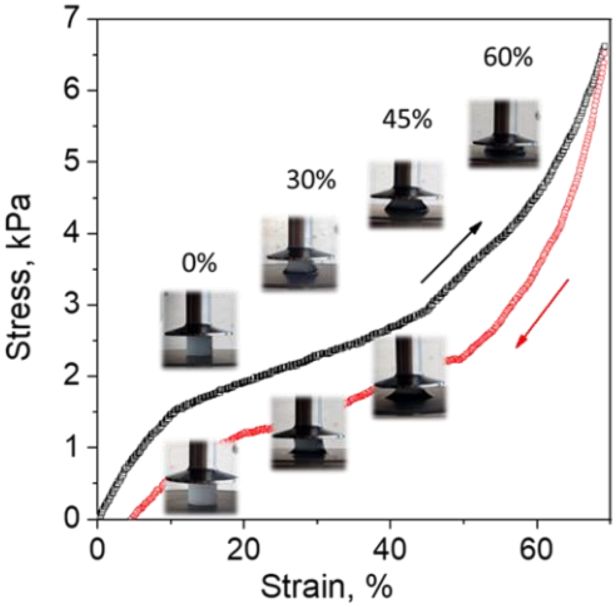

Figure S3. Strain-stress curve measured for CMC-PEG cryogen in the air and photos illustrated sample deformation and size recovery

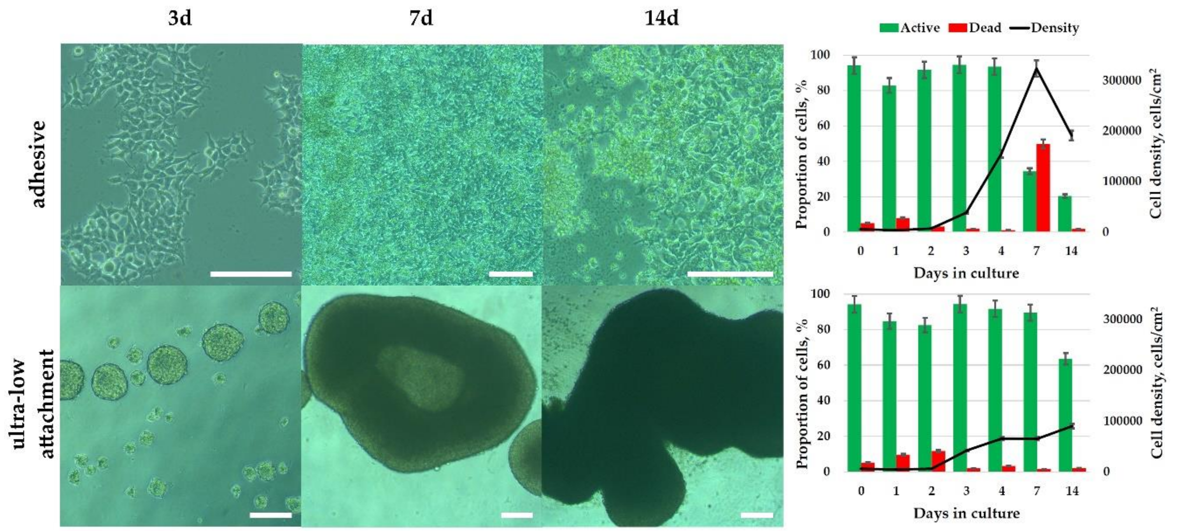

Figure S4. The results of flow cytometrical analysis and microscopic observation of human embryonic kidney cell line HEK-293T cultivated for 1, 2, 3, 4, 7 and 14 days in adhesive or ultra-low attachment plates. The cells were stained with H_2DCFDA to assess the mitochondrial activity, and DAPI to stain dead cells. The data is presented as a mean of three independent experiments. Standard deviations did not exceed 5%. The cells were imaged under a CKX41 inverted microscope (Olympus, Japan) equipped with phase-contrast optics. Scale bar – 200 μm.

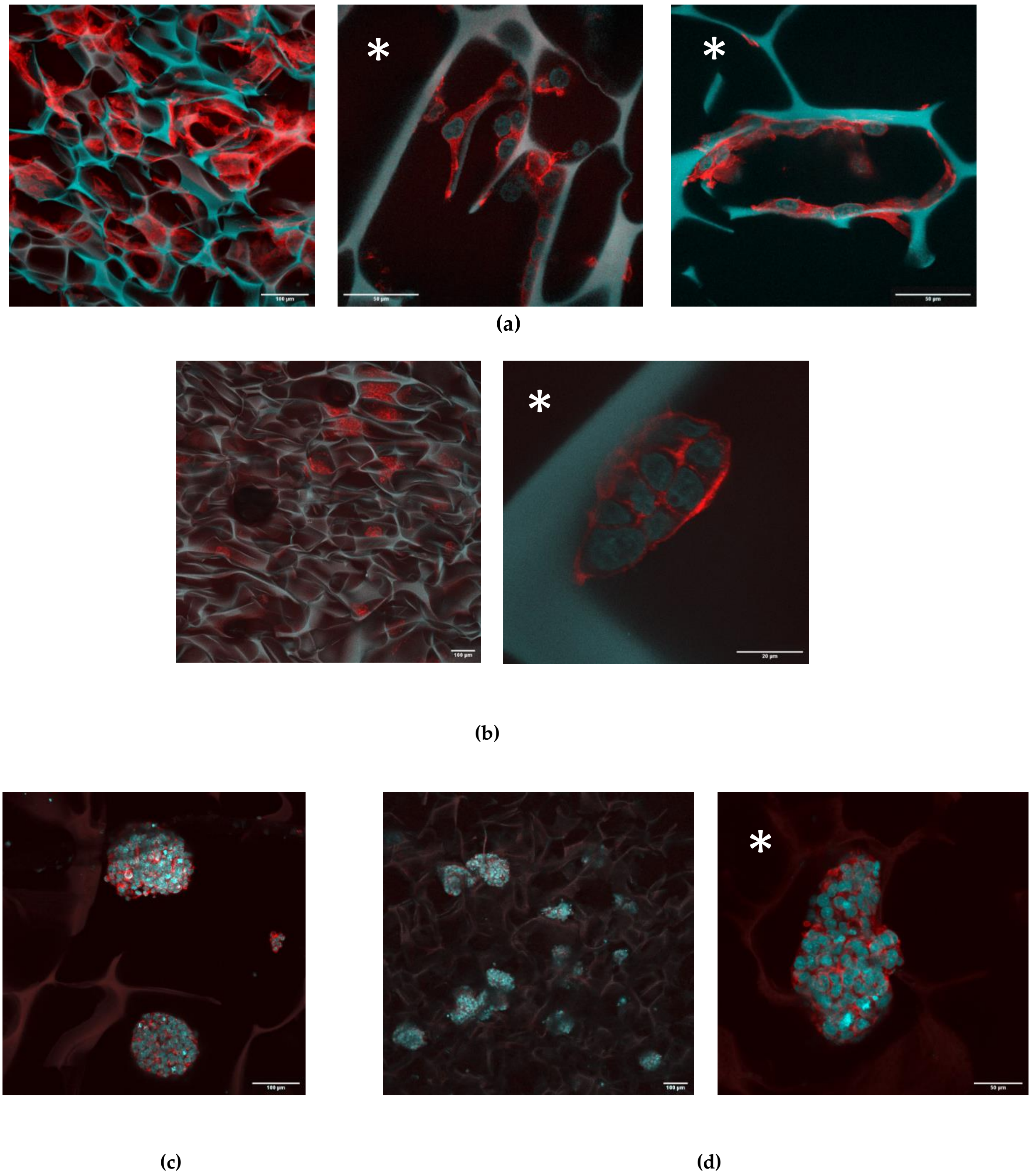

Figure S5 panels (a) through (d): panels marked * use a 50 µm scale bar (20 µm in one panel of (b)); the other panels use 100 µm. The panel text can't be read at this size.

Figure S5. Confocal laser scanning microscopy (CLSM) images of HEK-293T cells after 3 days of cultivation in CH-BD (a) and CH-PEG (b) cryogels and after 10 days of cultivation in CMC-PEG (c) and CMC-BD (d) cryogels. and chitosan (CH)-based cryogels (BDDGE:polymer ratio of 1:4) with HCT 116 cells after 3, 7, and 14 days of cultivation. Scale bar—100 µm or 50 µm for the images marked with asterisk.

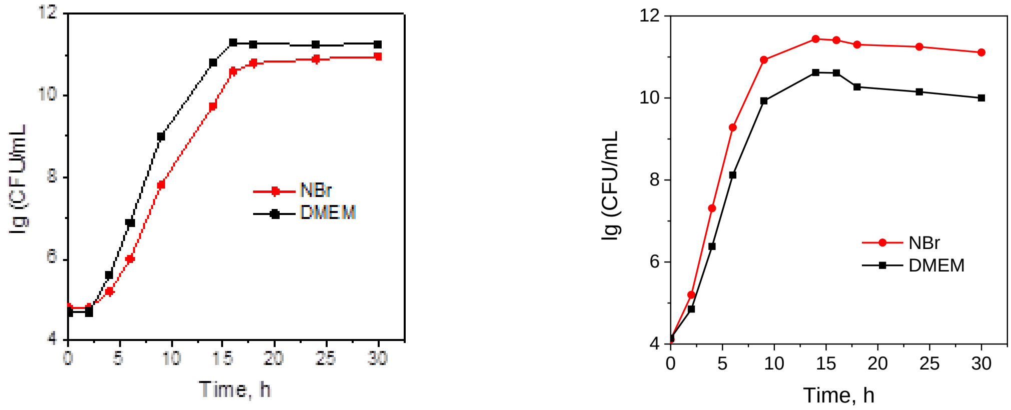

(**a**) (**b**)

Figure S6. Growth curves of *Pseudomonas fluorescens* 1574 (a) and *Staphylococcus aureus* ATCC 21027 (b) in Nutrient Broth (NBr) and DMEM cell culture medium.

Table S1. Monomer composition of carboxymethyl chitosan (CMC)

DA[1]	DS_{tot}[2]	N-DS[3]	O-DS[4]	Monomer composition			
				NH_2	NHR	NR_2	$NHCOCH_3$
0.25	1.49	0.29	1.20	0.46	0.29	0	0.25

[1] - Degree of acetylation, [2] - Degree of carboxyalkyl substitution (total), [3] - Degree of N-carboxyalkyl substitution, [4] - Degree of O-carboxyalkyl substitution

Table S2. Calculations of reagents quantities for the chitosan (CH) and carboxymethyl chitosan (CMC)**cryogels fabrication**

	Polymer	Cross-linker	Cross-linker : polymer molar ratio	3% polymer solution, g	Cross-linker, g*
CH-BD	Chitosan	BDDGE	1:4	5	0,07755
CH-PEG		PEGDGE	1:12	5	0.03834
CMC-BD	CMC	BDDGE	1:2	5	0.10136
CMC-PEG		PEGDGE	1:8	5	0.03759

***BDDGE used as 60% solution; PEGDGE Mn~ 500 g/mol, used as >99% solution**
